# Manganese-Enhanced T_1_ Mapping in the Myocardium of Normal and Infarcted Hearts

**DOI:** 10.1155/2018/9641527

**Published:** 2018-10-25

**Authors:** N. B. Spath, D. M. L. Lilburn, G. A. Gray, L. M. Le Page, G. Papanastasiou, R. J. Lennen, R. L. Janiczek, M. R. Dweck, D. E. Newby, P. C. Yang, M. A. Jansen, S. I. Semple

**Affiliations:** ^1^British Heart Foundation Centre of Cardiovascular Science, University of Edinburgh, Edinburgh, UK; ^2^Centre for Inflammation Research, University of Edinburgh, Edinburgh, UK; ^3^Edinburgh Preclinical Imaging, University of Edinburgh, Edinburgh, UK; ^4^GlaxoSmithKline, Gunnels Wood Road, Stevenage, Hertfordshire, UK; ^5^Department of Cardiology, Royal Infirmary of Edinburgh, Edinburgh, UK; ^6^Department of Cardiology, Stanford University, Stanford, CA, USA

## Abstract

**Background:**

Manganese-enhanced MRI (MEMRI) has the potential to identify viable myocardium and quantify calcium influx and handling. Two distinct manganese contrast media have been developed for clinical application, mangafodipir and EVP1001-1, employing different strategies to mitigate against adverse effects resulting from calcium-channel agonism. Mangafodipir delivers manganese ions as a chelate, and EVP1001-1 coadministers calcium gluconate. Using myocardial T_1_ mapping, we aimed to explore chelated and nonchelated manganese contrast agents, their mechanism of myocardial uptake, and their application to infarcted hearts.

**Methods:**

T_1_ mapping was performed in healthy adult male Sprague-Dawley rats using a 7T MRI scanner before and after nonchelated (EVP1001-1 or MnCl_2_ (22 *μ*mol/kg)) or chelated (mangafodipir (22–44 *μ*mol/kg)) manganese-based contrast media in the presence of calcium channel blockade (diltiazem (100–200 *μ*mol/kg/min)) or sodium chloride (0.9%). A second cohort of rats underwent surgery to induce anterior myocardial infarction by permanent coronary artery ligation or sham surgery. Infarcted rats were imaged with standard gadolinium delayed enhancement MRI (DEMRI) with inversion recovery techniques (DEMRI inversion recovery) as well as DEMRI T_1_ mapping. A subsequent MEMRI scan was performed 48 h later using either nonchelated or chelated manganese and T_1_ mapping. Finally, animals were culled at 12 weeks, and infarct size was quantified histologically with Masson's trichrome (MTC).

**Results:**

Both manganese agents induced concentration-dependent shortening of myocardial T_1_ values. This was greatest with nonchelated manganese, and could be inhibited by 30–43% with calcium-channel blockade. Manganese imaging successfully delineated the area of myocardial infarction. Indeed, irrespective of the manganese agent, there was good agreement between infarct size on MEMRI T_1_ mapping and histology (bias 1.4%, 95% CI −14.8 to 17.1 *P*>0.05). In contrast, DEMRI inversion recovery overestimated infarct size (bias 11.4%, 95% CI −9.1 to 31.8 *P*=0.002), as did DEMRI T_1_ mapping (bias 8.2%, 95% CI −10.7 to 27.2 *P*=0.008). Increased manganese uptake was also observed in the remote myocardium, with remote myocardial ∆T_1_ inversely correlating with left ventricular ejection fraction after myocardial infarction (*r*=−0.61, *P*=0.022).

**Conclusions:**

MEMRI causes concentration and calcium channel-dependent myocardial T_1_ shortening. MEMRI with T_1_ mapping provides an accurate assessment of infarct size and can also identify changes in calcium handling in the remote myocardium. This technique has potential applications for the assessment of myocardial viability, remodelling, and regeneration.

## 1. Introduction

With major and sustained advances in imaging techniques over the past 3 decades, magnetic resonance imaging (MRI), along with other advanced modalities such as positron emission tomography (PET), has become an essential element to noninvasive structural and functional cardiac imaging [1–3]. Current standard clinical methods use inversion recovery-delayed enhancement sequences after gadolinium-based contrast administration to image myocardial scar. This allows assessment of viability by assessing the transmural extent of myocardial scar and is used to predict prognosis, guiding the appropriateness of coronary revascularization [4], with excellent reproducibility [5]. Although an invaluable tool in viability assessment, the nonselective passive extracellular redistribution of gadolinium is unable to characterise and define viable myocardium directly [6]. Indeed, quantification by gadolinium delayed enhancement MRI (DEMRI) is subject to overestimation of acute infarct size due to tissue oedema [7]. It is also associated with imaging artefact and interpretation bias in challenging patient populations. Furthermore, whilst significant advances have enabled multiparametric MRI assessment of gadolinium distribution and dynamics to help determine aetiology [8], in practice the different patterns of late enhancement are neither completely specific nor sensitive for different forms of cardiac pathology where significant overlap is seen, as with aortic stenosis and cardiac sarcoidosis [9–11].

There has been increasing interest in a range of alternative contrast media [12] to broaden the capabilities and functional assessments of MRI. Manganese, a paramagnetic calcium analogue, enters active cardiomyocytes via voltage-gated calcium channels, increasing MRI-detectable T_1_ relaxivity [13]. As such, manganese-enhanced MRI (MEMRI) has the potential to quantify calcium influx and handling directly and to identify functional cardiomyocytes actively cycling calcium. Manganese-based contrast media can exist in either chelated (e.g. mangafodipir, manganese dipyridoxyl diphosphate, MnDPDP) or nonchelated forms (e.g. EVP1001-1, manganese gluconate with calcium gluconate [14]), and their uptake appears predominantly dependent on the activity of voltage-gated calcium channels during excitation-contraction coupling [15–17]. The different formulations of these manganese contrast media reflect different strategies to address adverse effects of manganese resulting from calcium-channel agonism which would otherwise be prohibitive to clinical use. Mangafodipir delivers manganese ions in the form of a chelated agent, similar to conventional MRI contrast agents, resulting in a lower effective circulating dose of manganese ions. Conversely, EVP1001-1 utilises coadministration of calcium, in the form of calcium gluconate, to negate toxicity. Both techniques have established safety and tolerability in clinical studies as well as efficacy in MRI contrast imaging.

Whilst short-term cardiac safety of intravenous MnCl_2_ has been suggested in a pilot study of 15 healthy volunteers at an equivalent molar dose [18], due to the risk of acute toxicity in cardiac patients, there is no expectation that MnCl_2_ be developed further for clinical utility. However, given that established clinical safety has been demonstrated for both EVP1001-1 and mangafodipir at doses required for cardiac MRI, there is widespread application for both mangafodipir and EVP1001-1, notably for cardiac imaging. Preclinical studies with mangafodipir and MnCl_2_ in healthy myocardium and EVP1001-1 in myocardial infarction models have described myocardial T_1_ shortening properties [19] and demonstrated favourable agreement with histological infarct assessment [20]. Moreover, recent preclinical studies have suggested that MEMRI can lead to better infarct discrimination and the identification of viable myocardium [21] as well as the ability to assess engraftment of myocardial stem cells [22].

Despite longstanding knowledge of the paramagnetic properties of manganese, the development and clinical translation of manganese contrast agents have been limited by early issues with toxicity and the subsequent widespread utility of gadolinium agents which have since dominated clinical use in the field. More recently, with concerns about neurological accumulation of some gadolinium agents [23–25], and as problems with acute manganese toxicity have been overcome, there is scope to revisit this agent with high potential in cardiac imaging.

Given the potential benefits of MEMRI, we aimed to compare myocardial enhancement using chelated and nonchelated manganese-based contrast media and to determine the contribution of calcium channels to their uptake. This study represents a novel head-to-head comparison of three manganese contrast agents, using T_1_ mapping to assess and compare their respective T_1_ shortening properties, utility and accuracy in quantifying myocardial infarction as compared with DEMRI (inversion recovery and T_1_ mapping) and histological analysis, and explore altered calcium handling in remodelling myocardium. This preclinical work is crucial to inform clinical translation and further development of the potential of MEMRI in myocardial viability assessment.

## 2. Methods

All studies were approved by the University of Edinburgh Animal Welfare and Ethical Review Body and were carried out in accordance with the UK Home Office Animals (Scientific Procedures) Act 1986. Male Sprague-Dawley rats (250–400 g, *n*=55) were purchased from Charles River Ltd (Haddington, UK) and housed, with free access to food and water, in the Central Bioresearch Services, University of Edinburgh for 7 days prior to use in the study.

### 2.1. Magnetic Resonance Imaging

All MRI experiments were performed using a 7T horizontal bore NMR spectrometer (Agilent Technologies, Yarnton, UK), equipped with a high-performance gradient insert (120 mm inner diameter), maximum gradient strength 400 mT/m. Rats were anaesthetised with 1.5–2% isoflurane (Zoetis Ltd., London UK) in oxygen/air (50/50, 1 L/min) with subsequent cannulation of the tail vein for drug/contrast agent administration. The animals were secured in a cradle (Rapid Biomedical GmbH, Rimpar, Germany). The heart rate, respiration rate, and rectal temperature were monitored (Model 1030 monitoring and gating system, Small Animal Instruments Inc. Stony Brook, NY, USA), with body temperature maintained at 37°C by a heat fan. A 72 mm quadrature volume coil was used for transmission with signal reception by a four-channel phased-array coil (Rapid Biomedical GmbH, Rimpar, Germany).

Scout images were taken to confirm correct positioning and to orientate 9 × 2 mm axial slices from the aortic valve annulus to the apex, perpendicular to the interventricular septum (short axis slices). The slice plan was carefully replicated between scans by ensuring the same slice plan methodology, which was agreed by two operators at the time of scanning to agree adequate orientation. Selection was then made of the mid-ventricle short axis slice for further interrogation with cine and the T_1_ mapping sequence. Long-axis cines and a short-axis stack were acquired (to allow left ventricular ejection fraction calculation), with cardiac-gated gradient echo imaging (TR = 1 × R-R interval; TE = 1.4 ms; flip angle = 15°; FOV = 50 × 50 mm^2^; matrix = 128 × 128; slice thickness = 1.5 mm).

T_1_ mapping for calculation of regional left ventricular myocardial T_1_ relaxation times was accomplished using a gradient-echo, cardiac-gated Modified Look-Locker Inversion recovery sequence (MoLLI) [26] whereby 14–20 images were acquired at unique inversion times (dependent on heart rate, ranging from approximately 0.20 to 3.00 s) with the TR_inversion_ > 3 × T_1_ of myocardium (TR_inversion_ > 4.50 s). Imaging readout was with a cardiac fast gradient echo (TR = 3.50 ms; TE = 1.77 ms; flip angle = 10°, matrix 128 × 128; ETL = 8; FOV = 50 × 50 mm^2^; in-plane resolution = 0.39 × 0.39 mm^2^; trigger delay = 1 × R-R; slice thickness = 2 mm; 8 signal averages) to compensate for respiratory motion.

### 2.2. Manganese-Enhanced MRI of Healthy Rat Myocardium

EVP1001-1 (SeeMore™, Eagle Vision Pharmaceuticals Corporation, Downingtown, PA, USA) was administered as an intravenous bolus at the manufacturer's recommended dosage of 22 *μ*mol/kg manganese over 3-4 min. Mangafodipir (Teslascan™, IC Targets AS, Oslo, Norway) was similarly administered as a bolus of 22 or 44 *μ*mol/kg manganese over 3-4 min. Manganese chloride solution (MnCl_2_) was prepared using MnCl_2_·4H_2_O (Sigma-Aldrich Ltd, Gillingham, UK) and sterile water (Sigma-Aldrich Ltd, Gillingham, UK), and 22 *μ*mol/kg manganese was administered over 3-4 min. All manganese contrast media were delivered in volumes of 2.2 mL/kg, diluted with 0.9% saline solution (Sigma-Aldrich Ltd, Gillingham, UK), to maintain the rate of manganese delivery constant between agents.

Diltiazem (Sigma-Aldrich Ltd, Gillingham, UK) diluted with 0.9% saline solution was infused at 100 *μ*mol/kg/min intravenously, increased to approximately 120–200 *μ*mol/kg/min until a satisfactory chronotropic response was achieved (reduction of >10% in heart rate) or the upper limit was reached. Infusion was commenced approximately 10 min prior to T_1_ mapping to ensure stable and adequate heart rate response. Control administrations consisted of a similar volume (8 mL/kg) of 0.9% saline over 180 min. Administration of all agents was followed by a further saline flush of 0.4 mL to ensure complete delivery to the circulation accounting for dead space in the fine-bore intravenous line. T_1_ mapping was performed in all animals at baseline and then at approximately 5, 20, 40, and 60 min after manganese contrast media administration, while cine imaging was performed in a cohort of animals at approximately 15, 30, and 50 min after contrast. The time intervals were determined by technical considerations relating to the length of time required for the T_1_ mapping sequences.

### 2.3. Myocardial Infarction Model

Rats were anaesthetised with isoflurane (5% in 1.5 L/min oxygen for induction), followed by intraperitoneal ketamine 100 mg/kg (Zoetis Ltd, London, UK) and medetomidine 1 mg/kg (Orion Pharma, Espoo, Finland) for maintenance anaesthesia. Buprenorphine 0.05 mg/kg (Alstoe Ltd, York, UK) was administered immediately before and 24 hours postoperatively for analgesia. Tracheal intubation was achieved under direct vision, and ventilation was maintained with a rodent ventilator (Harvard Apparatus Model 683, MA, USA, tidal volume 2.5 cm^3^, respiratory rate 60/min).

Myocardial infarction was induced as we have previously described [27]. Briefly, the skin was incised at the level of the left third and fourth ribs where the pectoral muscles were divided and retracted. Left lateral thoracotomy was then performed. With minimal handling, the pericardium was ruptured and the heart gently exteriorised from the thorax, and a nonabsorbable 5-0 ligature was placed around the left anterior descending coronary artery just above the bifurcation of the first diagonal and manoeuvred back into position. Before wound closure, a drain was inserted to assist with removal of air and fluid from the thorax. Once removed, the wound was then closed in three layers. Sham animals underwent identical surgery with pericardial rupture although the suture placed through the myocardium was not tightened to cause infarction. Animals were recovered with intraperitoneal atipamezole 0.1 mg/kg (Orion Pharma, Espoo, Finland) and extubated once spontaneous ventilation was established, housed at 30°C for 24 hours and given sterile sodium chloride 0.9% 0.01 mL/g fluid therapy subcutaneously. After 24 hours, normal housing conditions were resumed.

### 2.4. Myocardial Infarction Imaging

Three weeks postoperatively, rats first underwent DEMRI scanning, under isoflurane anaesthesia as described above. Scout images were taken to confirm correct positioning and to orientate 9 × 2 mm axial slices from the aortic valve annulus to the apex, perpendicular to the interventricular septum. Cine images were then acquired in long- and short-axis views as outlined above. Standard DEMRI inversion recovery was performed using gadolinium complex (gadobendate dimeglumine, Bracco S.p.A, Milan, Italy) with 0.5 mmol/kg administered intravenously via slow injection into the tail vein over 1-2 min. Standard inversion recovery prepared imaging with myocardial nulling was performed 10 min following injection (inversion recovery gradient echo, TI = 2.3 × R-R (typical R-R 150–200 ms), TR ≈ 500 ms; TE = 1.6 ms; flip angle = 90°; FOV = 50 × 50 mm^2^; matrix = 128 × 128). Due to information gained from the healthy animal data with the manganese contrast agent experiments (Section 3) as well as technical considerations (i.e., pulse sequence duration), DEMRI T_1_ mapping was performed at 20 min following contrast injection upon completion of inversion recovery, at the maximal infarct slice as defined by the cine images (MoLLI: TR > 4.5 sec; TE = 1.7 ms; flip angle = 10°; matrix = 128 × 128; ETL = 8; FOV = 50 × 50; 20 time points; trigger delay = 1 × R-R; slice thickness = 2 mm; 8 signal averages). Animals were allowed to recover following the scan.

MEMRI was performed 48 h after the DEMRI protocol. Scout images were taken followed by a single short-axis cine at the maximal infarct slice, using the method outlined above. MEMRI T_1_ mapping was then achieved using one of the two manganese-based MRI contrast media being developed for clinical use; EVP1001-1 (*n*=8) and mangafodipir (*n*=9) at doses of 22 and 44 *μ*mol/kg, respectively, administered via slow intravenous injection into the tail vein over 1-2 min. T_1_ mapping was performed at the maximal infarct slice (EVP1001-1, 20 min after injection; mangafodipir 40 min after injection) defined by the DEMRI scan acquired in the first imaging session. The doses selected of EVP1001-1 and mangafodipir and the timings of the T_1_ mapping sequences after manganese contrast agent administration were informed by the healthy animal data to ensure similar degrees of T_1_ enhancement and therefore sensitivity to detect myocardial viability. Finally, a cine acquisition in the short axis at the maximal infarct slice was repeated following contrast injection. Animals were allowed to recover following the scan.

**Figure 1:**
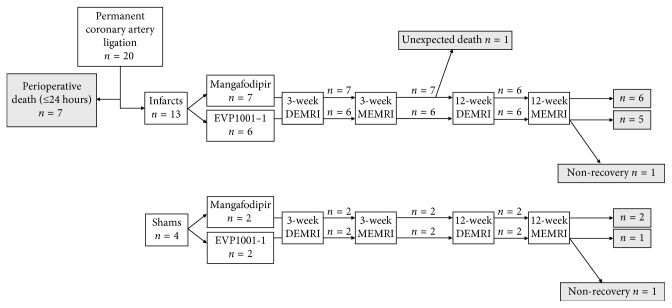
Myocardial infarction experimental protocol. Flow chart detailing timing of surgery and imaging with different contrast agents.

At 12 weeks after surgery, DEMRI and MEMRI (48 h apart) were repeated using the identical protocols described above. Animals received the same manganese contrast agent at both time points. MRI parameters were unchanged with the exception of an increased FOV (55 × 55 mm) on account of growth of the animals. After the second MEMRI scan, animals were culled by exsanguination by femoral puncture under anaesthesia for tissue harvest.  Figure 1 displays a flow chart summarising the imaging of the myocardial infarction cohort.

### 2.5. Pathology

Hearts were fixed by immersion in 4% paraformaldehyde for 24 hours before being transferred to 70% ethanol and processed to paraffin wax for sectioning thereafter. Serial 5 *µ*m sections were taken at intervals in the short axis from apex to base, corresponding to MRI short axis T_1_ mapping data. Staining was performed with Masson's trichrome (MTC) to delineate areas of collagenous fibrosis, staining infarct blue, and noninfarct purple, before mounting for computer-aided analysis. Slides were scanned at 20x magnification on a Zeiss Axio Scan Z1 (Carl Zeiss AG, Oberkochen, Germany) with infarct size calculated as a percentage of total left ventricular area at the comparable maximal infarct slice defined by MRI. Automated tissue detection was conducted using Tissue Studio v2.4 (Definiens AG, Munich, Germany) as follows: a training set of 4 images was automatically segmented, and segments within three 50 × 50 *µ*m regions comprising remote myocardium, infarct, and cross-over regions from each training image (12 regions in total) were manually classified as “Normal myocardium,” “Collagen,” and “nontissue.” These manual classification samples were used to train the software's automated classification algorithm which was then applied to all images in the dataset. Automated detection produced pixel counts and areas for the ROIs within the left ventricle, from which percentage infarct area at maximal infarct slice was calculated.

### 2.6. Image Analysis

Quantitative analysis of manganese accumulation was achieved by calculation of regional T_1_ relaxation times before and after administration of manganese contrast media. The 14–20 images at unique inversion times were exported offline and combined to generate T_1_ maps using commercially available software (CVI^4.2®^, Circle Cardiovascular Imaging, Calgary, Canada) using three-parameter nonlinear curve fitting as previously described [15].

During the lengthy *in vivo* experiments, it was noted that there was an approximate ±10% variation in the measurement of T_1_ values in healthy myocardium and skeletal muscle before the administration of manganese contrast agents. In an attempt to compensate for these fluctuations in T_1_ measurements, myocardial T_1_ values were normalized to the T_1_ of skeletal muscle, where it was noted that the T_1_ values did not vary over the time course of the contrast agent infusion (as detailed in Section S1 of Supplementary Materials). Final normalized T_1_ maps were then generated in Matlab (MathWorks, Inc., USA) with normalized myocardial T_1_ values obtained from regions of interest (ROIs) drawn on the left ventricle. Change in T_1_ between the baseline myocardial T_1_ and the myocardial T_1_ at each time point was then calculated (ΔT_1_). Infarct was defined as 2x standard deviations of infarcted and remote myocardial T_1_ with an averaged intermediate value representing borderzone myocardium.

### 2.7. Statistical Analysis

Data are presented as mean ± standard deviation unless otherwise stated. Comparison of the time course curves between the saline (control) and diltiazem-infused groups for each of the 22 *μ*mol/kg MnCl_2_, 22 *μ*mol/kg EVP1001-1, and 44 *μ*mol/kg mangafodipir was performed using a two-way analysis of variance (ANOVA) with post hoc multiple comparison Bonferroni tests for individual time points (compared to baseline). For infarct quantification, DEMRI inversion recovery, DEMRI T_1_ mapping, MEMRI T_1_ mapping, and MTC assessments were compared using Wilcoxon signed-rank test (2-tailed), with post hoc multiple comparison Bonferroni testing. Bland–Altman plots were used to assess agreement between DEMRI inversion recovery, DEMRI T_1_ mapping, MEMRI T_1_ mapping and MTC, and Pearson correlation tested for relationship between changes in remote T_1_ between early and late time points (ΔT_1_, dependent variable) and ejection fraction (independent variable). Statistical analysis was performed using GraphPad PRISM (v.7.0, GraphPad Software, Inc., La Jolla, CA, USA). Statistical significance was taken as two-sided *P*<0.05.

## 3. Results

### 3.1. Comparative and Calcium-Channel Dependency of MEMRI Contrast Media

**Figure 2:**
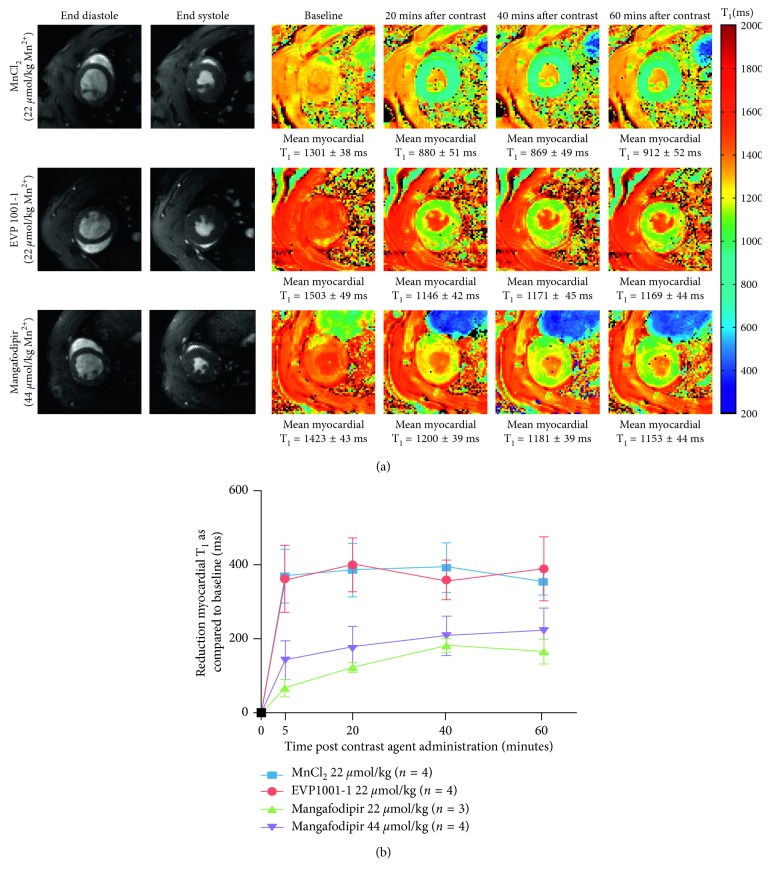
T_1_ shortening of manganese contrast media over time. (a) Normalized T_1_ maps acquired subsequent to infusion of MnCl_2_, EVP1001-1, and mangafodipir at 20 minute intervals up to 60 minutes, with associated gradient echo cine images in end-diastole and end-systole. MnCl_2_ (22 *μ*mol/kg), EVP1001-1 (22 *μ*mol/kg) or mangafodipir (44 *μ*mol/kg) was administered intravenously to isoflurane-anaesthetised healthy rats over 3-4 minutes. Rats were simultaneously administered an infusion of 8 mL/kg 0.9% saline over 3-4 minutes. Note the superior degree of T_1_ shortening with MnCl_2_, and EVP1001-1 at half the molar dosage of manganese as compared with mangafodipir (T_1_ reduction of 421.3 ms and 357.9 ms from baseline with MnCl_2_ and EVP1001-1 compared with 222.7 ms with mangafodipir). (b) Reduction in mean left ventricular T_1_ values over 60 minutes with EVP1001-1 and mangafodipir. MnCl_2_ (22 *μ*mol/kg; blue), EVP1001-1 (22 *μ*mol/kg; red), mangafodipir (22 (green) or 44 (purple) *μ*mol/kg) was administered to rats (*n*=4 per group) over 3-4 min. Error bars represent standard deviations from time points where measurements were recorded (*n*=4 at each time point). Two-way ANOVA confirmed a dependence of mean myocardial T_1_ shortening between each of the contrast agents (*P* < 0.0001).

Thirty-one animals underwent experiments with the manganese contrast agent with a concurrent infusion of either 0.9% saline or diltiazem at a median age of 84 ± 19 days, with a median weight of 356 ± 45 g. Four animals (two control and two with diltiazem) were excluded as venous access was compromised resulting in unpredictable contrast agent administration. MnCl_2_, EVP1001-1, and mangafodipir altered T_1_ relaxivity values evident with T_1_ mapping (Figure 2(a)). Mean shortening of myocardial T_1_ values was greater with EVP1001-1 as compared with similar concentrations of mangafodipir but commensurate to those observed with MnCl_2_ (Figure 2(b)). Peak changes in T_1_ values were obtained by 20 min with EVP1001-1 and MnCl_2_, with persistent T_1_ shortening at 60 min. The magnitude of T_1_ shortening was dose-dependent for mangafodipir with little change between 40 and 60 min time points (Figure 2(b)). Overall T_1_ shortening with MnCl_2_, EVP1001-1, and mangafodipir (all 22 *μ*mol/kg) at 20 min were 29.4 ± 5.1%, 28.0 ± 4.4%, and 8.5 ± 4.2%, respectively. To improve the degree of T_1_ shortening with mangafodipir, the dosage of manganese administered was increased to 44 *μ*mol/kg resulting in a T_1_ shortening of 12.8 ± 3.4% at 20 min which increased to 15.0 ± 2.9% at 40 min. MnCl_2_ and EVP1001-1 values at 40 min after administration were unchanged.

Pre-treatment with a calcium-channel antagonist (diltiazem) inhibited MEMRI-induced T_1_ shortening (Table 1). This inhibition was similar between all agents with MnCl_2_ experiencing a maximal mean reduction of 30% in T_1_ shortening between the 5 and 60 min time points, while EVP1001-1 and mangafodipir experienced reductions of up to 43% and 32%, respectively. There were significant differences between the degree of myocardial T_1_ shortening due to each manganese contrast agent in the presence of diltiazem as measured by two-way ANOVA (MnCl_2_ *P*<0.0004, EVP1001-1 *P*<0.0001 and mangafodipir *P*=0.044).

### 3.2. Left Ventricular Function

All contrast agents were well tolerated across all animal groups. Following administration of gadolinium and EVP1001-1, a transient increase in the respiratory rate was observed, which was self-limiting and resolved within 1 minute. This was seen in healthy animals, shams, and infarcted animals. The present study was not designed to assess safety and tolerability, and no further adverse effects were observed.

Healthy myocardial left ventricular ejection fraction was measured before and after manganese contrast agent administration to assess for discernible myocardial depression. The mean LVEF (±standard deviation) and the mean difference in LVEF at each time point from the cohorts of healthy rats administered with each of the manganese contrast agents with concurrent diltiazem (or 0.9% saline control) infusion are shown in Table 2.

No difference was noted between the mean difference in LVEF between baseline and the time points between mangafodipir 22 *μ*mol/kg and mangafodipir 44 *μ*mol/kg (*P*=0.78) by two-way ANOVA indicating that the higher mangafodipir dosage was well tolerated. There were significant differences detected between the mean difference in LVEF with each of the contrast agents in the presence of diltiazem: MnCl_2_ 22 *μ*mol/kg (*P*<0.001 and significant (*P*<0.05) at the 15 min and 50 min time points on multiple comparison); EVP1001-1 (*P*=0.02) and mangafodipir 44 *μ*mol/kg (*P*=0.04). This may potentially indicate that in the presence of depressed LVEF the manganese contrast agents at the dosages used may further compromise myocardial contractility; however, further study would be required beyond this preclinical study with low numbers of animals. There was no change in left ventricular ejection fraction following manganese administration with any of the agents in the saline control animals. These data support the assertion that there is minimal change in left ventricular myocardial function in healthy animals with the dosages used in this study. In the infarct group, left ventricular function was assessed at the maximal infarct slice by fractional area change (single slice only due to concern over prolonged anaesthetic risk) before and after manganese-based contrast media administration to assess for discernible myocardial depression. There was no change in fractional area change at this slice following administration of either EVP1001-1 or mangafodipir at early (0.1 ± 1.5%, *P*=0.82 and 0.1 ± 2.2%, *P*=0.88) or late (0.2 ± 1.2%, *P*=0.74 and 0.3 ± 1.8%, *P*=0.70) imaging time points, respectively.

### 3.3. Effect of T_1_ Relaxivity of Contrast Media

**Figure 3:**
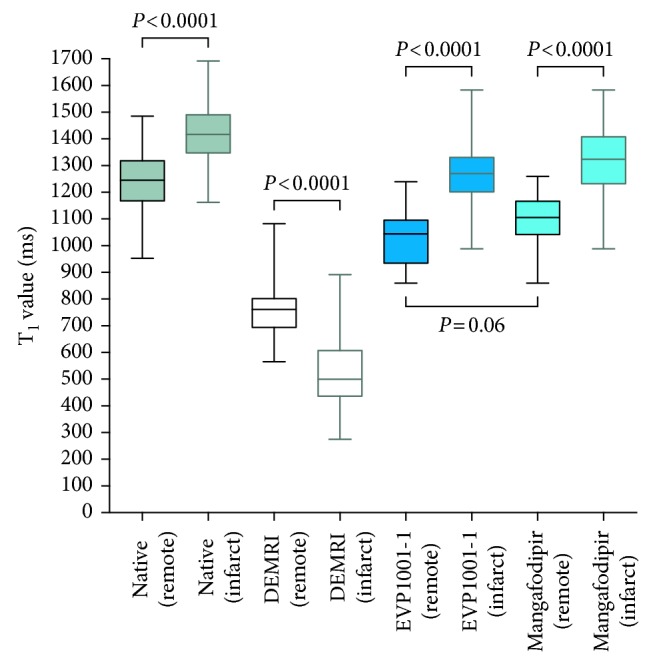
Comparison of the effects of contrast agents on T_1_ relaxivity. Mean T_1_ is significantly different between remote and infarcted areas of myocardium for all agents and native T_1_ mapping (all *P*<0.0001). Remote myocardial T_1_ between manganese contrast agents was comparable (*P*=0.064).

On comparison of the effect of different contrast agents on T_1_ relaxivity, the difference in mean T_1_ between infarcted and remote myocardium was highly significant across all contrast agents as well as native T_1_ mapping. Remote myocardial mean T_1_ was similar between both mangafodipir and EVP1001-1 (Figure 3).

### 3.4. Viability Assessment after Myocardial Infarction

Eighteen animals underwent successful surgery (14 permanent coronary artery ligation surgery, 4 sham surgery) at a median age of 58 ± 7 days, with a median weight of 262 ± 52 g. One animal in the surgical cohort died unexpectedly 11 days after surgery, resulting in 17 animals completing the experimental imaging protocol. Another animal died unexpectedly 31 days after surgery, with large myocardial infarction, a likely substrate for ventricular arrhythmia as previously observed [28], and 2 animals failed to recover from MRI at the late time point.

Three weeks following surgery, all animals in the infarct cohort had left ventricular impairment with anterior wall akinesis and wall thinning associated with a reduced left ventricular ejection fraction (42.2 ± 8.1 versus 68.9 ± 9.4% in sham animals, *P*<0.001). Myocardial infarction was also associated with higher left ventricular end diastolic volume (1.0 ± 0.2 versus 0.7 ± 0.1 mL, *P*=0.02) and mass (0.7 ± 0.1 versus 0.6 ± 0.04 g, *P*=0.03). There were no differences in left ventricular function nor volume between animals administered mangafodipir or EVP1001-1 (left ventricular ejection fraction, 41.49 ± 9.86 and 43.10 ± 6.44%, respectively, *P*=0.76; left ventricular end diastolic volume, 1.02 ± 0.21 versus 0.83 ± 0.07 mL, respectively, *P*=0.18).

**Figure 4:**
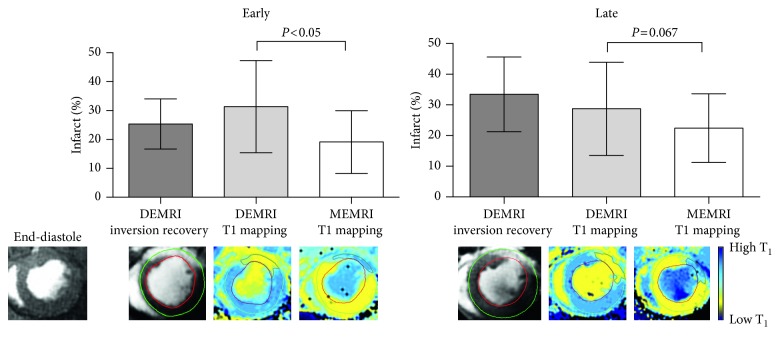
Comparison of DEMRI and MEMRI infarct quantification by T_1_ mapping. Mean infarct size as a percentage of left ventricular myocardium at maximal infarct short-axis slice in rats with DEMRI and MEMRI T_1_ mapping at two time points; early post-MI (3 weeks, *n*=13, left panel) and late post-MI (12 weeks, *n*=12, right panel). Infarct size as assessed by MEMRI T_1_ mapping is significantly lower than DEMRI T_1_ mapping at 3 weeks (*P*<0.05), a result which is attenuated at 12 weeks (*P*=0.067). Error bars represent standard deviation. Example T_1_ maps with delayed enhancement and gradient echo cine images are shown for one animal.

After surgery, infarct size at the maximal infarct slice was smaller when assessed by MEMRI than DEMRI at 3 weeks (17.4 ± 8% versus 28.5 ± 13%, *P*<0.05), although the differences were less marked by 12 weeks (20.4 ± 9% versus 28.6 ± 8%, *P*=0.067, Figure 4).

**Figure 5:**
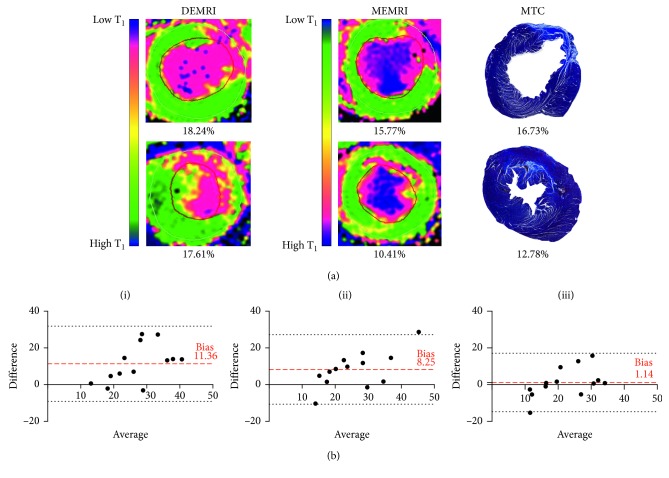
DEMRI versus MEMRI vs MTC. (a) Comparison of magnetic resonance imaging and histological quantification of infarct size. Infarct size as a percentage of left ventricular myocardium at maximal infarct short-axis slice by DEMRI and MEMRI T_1_ mapping and histologically with MTC. Note the inverted T_1_ colour map configuration between DEMRI T_1_ mapping and MEMRI T_1_ mapping, calibrated to define infarct (pink) and remote (green) myocardium with intermediate values (yellow). (b) Bland–Altman plots showing differences between DEMRI inversion recovery (A), DEMRI T_1_ mapping (B), and MEMRI T_1_ mapping (C) for each rat heart. The average difference (bias) between the measurements is shown (dashed lines) ±2 × SD (dotted lines) for all three modalities.

At 12 weeks, DEMRI inversion recovery, DEMRI T_1_ mapping, and MEMRI T_1_ mapping of infarct size all correlated independently with histologically quantified infarct size by MTC (all *P*<0.05). However, unlike manganese, gadolinium-based assessments tended to overestimate infarct size by around 10% (DEMRI inversion recovery, bias 11.36%, 95% confidence intervals −9.11 to 31.82, *P*=0.002; DEMRI T_1_ mapping, bias 8.25 %, 95% confidence intervals −10.7 to 27.2, *P*=0.008; MEMRI T_1_ mapping, bias 1.14 %, 95% confidence intervals −14.8 to 17.1, *P*=0.735; with post hoc Bonferroni multiple comparisons for *P*<0.05, Figures 5(a) and 5(b)).

**Figure 6:**
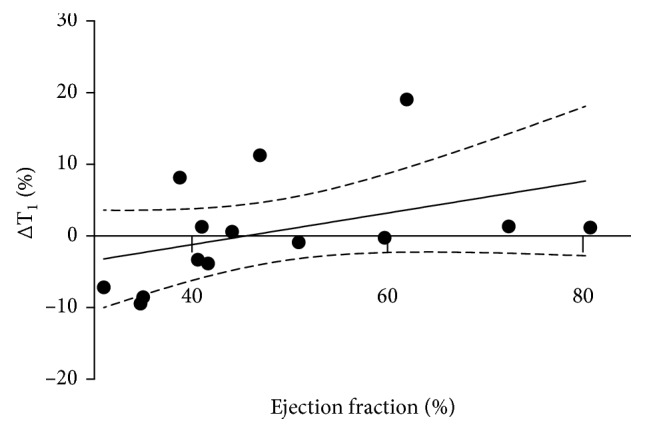
∆T_1_ in remote myocardium over time versus ejection fraction. Correlation of change in remote myocardial T_1_ relaxivity between early and late time points with ejection fraction at 12 weeks after surgery. There is a significant correlation between ejection fraction and T_1_ reduction between early (3 week) and late (12 week) time points (*r*=0.61, *P*=0.022). Standard error of the mean shown as dashed black line.

There was an inverse correlation between ejection fraction and remote myocardial ∆T_1_ (*r*=−0.61, *P*=0.022; Figure 6) with greater reduction in remote myocardial T_1_ at 3 months with increasing severity of left ventricular impairment. Myocardium remote from the site of infarction (mean T_1_ of noninfarcted myocardium) appeared to have lower mean MEMRI T_1_ mapping values at late (12 week) compared with early (3 week) time points in animals with the largest infarcts by ejection fraction, but this was not statistically significant (mean ∆T_1_ −8.39 ± 0.66%, *P*=0.4, *n*=3; sham animals with preserved left ventricular ejection fraction mean ∆T_1_ 7.19 ± 5.93%, *P*=0.7, *n*=3).

## 4. Discussion

The present study applies myocardial T_1_ mapping to manganese-enhanced MRI, in healthy myocardium in addition to remote myocardium after infarction. This novel combination of imaging techniques has been employed to directly compare two distinct manganese contrast agents with conventional DEMRI in the assessment of viability by infarct size, as well as examine altered calcium handling in remodelling myocardium over time, building on previous pilot data in myocardial infarction. This work was designed as a precursor to clinical translation of intramyocardial contrast imaging, for development of this promising field within cardiac MRI which has potential to improve accuracy of myocardial viability assessment, improve understanding of pathophysiology, and monitor response to therapy in different forms of cardiomyopathy.

We have demonstrated that MEMRI causes an ionic, concentration, and calcium channel-dependent shortening of myocardial T_1_ values. We have further shown that MEMRI T_1_ mapping provides a better estimate of infarct size than DEMRI using both inversion recovery and T_1_ mapping and correlates with left ventricular remodelling within the remote myocardium following myocardial infarction. We conclude that MEMRI holds major potential for the assessment of myocardial viability, dysfunction, and regeneration with a wide range of clinical applicability.

### 4.1. Chelation, Concentration, and Calcium-Channel Dependence

Biotransformation of MnDPDP occurs by dephosphorylation and simultaneous transmetallation with zinc facilitating MRI-detectable intracellular manganese uptake, as demonstrated *in vitro* where transmetallation with zinc occurs rapidly, almost to completion, within 1 minute of incubation in human serum [29]. These findings have been reinforced in subsequent animal [30, 31] and human studies [32, 33]. In the present study, chelated (mangafodipir) and nonchelated (MnCl_2_ and EVP1001-1) manganese contrast media were compared in healthy myocardium. Intracellular T_1_ shortening properties of manganese were clearly demonstrated with a reduction in myocardial T_1_ values of 29.4 ± 5.1%, 28.0 ± 4.4% and 12.8 ± 3.4%, compared with baseline values, with 22 *μ*mol/kg MnCl_2_ and EVP1001-1 at 20 min, and 44 *μ*mol/kg mangafodipir at 40 min, respectively. The paramagnetic performance of manganese when administered as EVP1001-1 was highly comparable to that of MnCl_2_, with rapid increase in T_1_ relaxivity over time achieving close to maximal relaxivity by 5 min (Figure 2(a)). This correlation is expected given intravenous administration of nonchelated manganese ions in both cases. This effect on T_1_ shortening was sustained at 60 min and an optimal imaging time point of 20 min was adopted to allow for variation in administration dynamics and utilised in the infarcted myocardium MEMRI experiments. A less marked increase in relaxivity was observed with mangafodipir. This is likely to be due to the need for manganese to become unchelated from the DPDP ligand as above. To achieve reduction in T_1_ comparable with the nonchelated preparations, the dose of mangafodipir was doubled to 44 *μ*mol/kg to obtain similar reductions in myocardial T_1_ (Figure 2(b)). T_1_ relaxivity continued to increase over the measured time period although appeared to begin to plateau from 40 min after administration. An imaging time point of 40 min was selected as a compromise between practicability and allowing adequate time for unchelation of manganese to achieve sufficient intracellular uptake and therefore provide adequate T_1_ shortening. Inhibition of intracellular manganese uptake was evident from a consistently reduced T_1_ shortening observed with MnCl_2_, EVP1001-1, and mangafodipir when coadministered with diltiazem (Table 1). A benzothiazepine, diltiazem binds to L-type calcium channels at cardiac myocytes and decreases myocardial contractility [8]. Coadministration with manganese-based contrast agents serves to assess manganese uptake in myocardium during calcium-channel inhibition. Myocardium in animals pretreated with diltiazem showed reduction in mean shortening of myocardial T_1_ values with both clinical-grade agents, but the magnitude of inhibition was greater with EVP1001-1 compared with similar concentrations of mangafodipir. Due to the superior T_1_ shortening with EVP1001-1 at lower doses, there are potentially greater differences between the diltiazem and saline infused animals. These data reinforce the understanding that intracellular manganese uptake is dependent on both L-type voltage-gated calcium channel as well as sodium/calcium exchanger activity, as previously demonstrated [34].

### 4.2. MEMRI in Myocardial Infarction

In myocardial infarction, both DEMRI inversion recovery and T_1_ mapping consistently overestimated infarct area 3 weeks after surgery in comparison with MEMRI T_1_ mapping. Both DEMRI and MEMRI modalities correlated with histopathological infarct quantification by MTC. However, infarct quantification was similar for histopathology and MEMRI T_1_ mapping, whereas DEMRI consistently overestimated infarct size. Contrast imaging of acute myocardial infarction with DEMRI inversion recovery is well established to overestimate infarct size due to pathologically expanded extracellular space and myocardial oedema. This finding has been observed with preclinical data from swine ischaemia-reperfusion injury indicating discrepancy between DEMRI inversion recovery (both *in vivo* and *ex vivo*) and histological infarct size at 6 h, resolving at 7 days [35]. In clinical studies, imaging too early after infarction results in enhancement of salvaged as well as infarcted myocardium [5] and some degree of myocardial oedema remains and is stable for 7 days following myocardial infarction, reducing at 14 days and near-normalising at 6 months [36]. MEMRI mechanistically circumvents the uncertainty of myocardial oedema as it acts as a specific intracellular agent tracking cardiomyocytes with functional calcium handling. Furthermore, permanent arterial occlusion models, as used in the present surgical protocol, result in substantially less myocardial interstitial oedema than ischaemia-reperfusion models, even at 24 h [37]. In the present study, gross myocardial oedema is therefore unlikely to persist at 3 weeks, implicating other factors to account for overestimation of infarct size by DEMRI techniques. We hypothesise that dual enhancement with both gadolinium and manganese may occur in areas of injured myocardium where there is residual calcium transport functionality. Early clinical work has explored MEMRI T_1_ mapping as a technique to define viability in this way using mangafodipir, demonstrating manganese enhancement in myocardium remote to the infarcted region, 3-4 weeks after infarction [38]. Whilst MnCl_2_ carried significant risk of adverse events in cardiac patients which prohibits further clinical development, mangafodipir and EVP1001-1, both having established clinical safety and tolerability, are now primed for clinical applications in cardiac imaging. The clinical significance of viable myocardium defined in this way is as yet unknown and underscores the need for robust clinical trials in this field.

### 4.3. Detection of Altered Calcium Handling with MEMRI

T_1_ mapping of remote myocardium was compared over time following myocardial infarction allowing time for left ventricular remodelling. Changes in T_1_ values between early and late time points were compared for each animal. Despite variability between animals, an inverse correlation was observed between ejection fraction and change in T_1_ value for all animals, including shams. The significance and precise mechanisms underlying this preliminary finding are unconfirmed as there are contradictory data on activity of voltage-gated L-type calcium channels in heart failure. L-type calcium channels in remodelling remote myocardium may have a greater propensity to remain open for longer, on account of prolongation of the plateau phase of the cardiac action potential, as compared with uninjured myocardium resulting in greater relative manganese uptake [39]. A study analysing cardiomyocytes from failing human myocardium observed enhancement of single L-type calcium-channel activity compared with nonfailing control myocardium, demonstrating both increased availability and open probability [40]. The data from the present study indicate potential for MEMRI to characterise disordered calcium handling in failing myocardium, but this requires further exploration in clinical translational studies.

### 4.4. Challenges of the Preclinical Model

The present study has been designed as a proof-of-concept study of MEMRI in ischaemic cardiomyopathy to inform clinical translation of this imaging modality in ischaemic heart disease as well as other forms of cardiomyopathy. The aim was to assess the application of manganese in noninfarcted myocardium undergoing remodelling following infarction rather than specific dynamics of the infarct region acutely after infarction. Therefore, a permanent coronary artery ligation model was used over an ischaemia-reperfusion model. There are several aspects that are specific to preclinical MR imaging of rodents which do not apply to clinical imaging which are relevant. Despite the excellent spatial resolution of the T_1_ mapping sequence used in this study, the small myocardial volume in conjunction with heart rates in excess of 350 beats per minute, obligate free-breathing acquisition with respiratory rates in excess of 40 breaths per minute (with a high degree of variability in both these parameters) provide significant challenges resulting in prolonged T_1_ mapping sequences (11–13 minutes) and the margin for error is consequently much narrower than in a clinical equivalent. This constraint resulted in the practical requirement to select one slice representative of the myocardial infarction, rather than acquire a full T_1_ map short-axis stack. The potential for sampling error between scans was minimized by careful adjudication by two experienced operators at the time of scanning to agree adequate orientation and replicate slice planning methodology, as described above. Whilst unable to guarantee maximal infarct slice, this ensures equivalent slice comparison between scans.

The use of T_1_ mapping removes the issue of timing in correct nulling, which is made problematic due to the animal-specific issues above. In the imaging of an infarct, it is possible to select an inversion time following manganese administration whereby the infarct is nulled and the myocardium enhances, in an opposite fashion to DEMRI with gadolinium. Inversion recovery imaging with these nulling techniques is easily degraded by artefact of irregular heart rate or breathing and accurate conclusions are highly dependent on the quality of the nulling. Moreover, where more diffuse myocardial processes are concerned, such as remote remodelling in ischaemic cardiomyopathy, T_1_ mapping offers the ability to quantify the graduation of T_1_ across all regions of myocardium. Finally, in clinical MR imaging, a motion correction algorithm is applied to the T_1_ mapping sequence. This algorithm is not available to us in the preclinical setting. These technical factors, unique to the preclinical nature of this study, necessitated the use of normalization of T_1_ values and underscore the need for clinical translation.

### 4.5. Clinical Perspectives

What future diagnostic and therapeutic possibilities does manganese-enhanced cardiac MRI offer the clinician? The prospect of intracellular myocardial tissue characterisation is novel and has far reaching potential. The present study demonstrates unique description of myocardial infarction and viability through calcium handling which has exciting applications in development of novel therapies in myocardial infarction, recently explored in preclinical ischaemia-reperfusion assessing myocardial regeneration with stem cell therapy [13] and clinical work establishing safety and tolerability of chelated manganese contrast agents in ischaemic heart disease [23]. Beyond myocardial infarction, we have demonstrated potential to detect altered calcium handling noninvasively and scope for earlier detection and quantification of cardiomyopathy. The present preclinical study highlights the need for clinical translation of these agents, where image acquisition is vastly superior, in a patient population which accurately represents the substrate for disease underlying the pathology.

## 5. Conclusion

The present study demonstrates the utility of MEMRI with two distinct agents, chelated and nonchelated manganese, as a noninvasive imaging modality which can accurately quantify viable myocardium. Furthermore, these data indicate an ability to detect and quantify altered calcium handling in the remodelling remote myocardium. This novel technique has potential to actively quantify viable myocardium, rather than inferring viability using infarct extent as a surrogate. Furthermore, calcium-handling dysfunction observed in a wide range of cardiomyopathies and heart failure syndromes eludes current noninvasive investigation, an application where MEMRI holds great promise. Clinical translation of the work presented here is an essential next step.

## Figures and Tables

**Table 1:** Effect of diltiazem on manganese-induced T_1_ shortening.

Time point (minutes)	Baseline T_1_ (ms)	Mean T_1_ shortening (ms)			
		5	20	40	60
MnCl_2_ + saline	1286 ± 42	366 ± 72	382 ± 72	389 ± 67	351 ± 35
MnCl_2_ + diltiazem (*n*=3)	1180 ± 93	290 ± 52	288 ± 71	276 ± 56	274 ± 63
EVP1001-1 + saline	1265 ± 94	354 ± 91	397 ± 73	352 ± 54	383 ± 87
EVP1001-1 + diltiazem	1375 ± 38	209 ± 27^*∗*^	225 ± 57^*∗*^	211 ± 49	226 ± 40^*∗*^
Mangafodipir + saline	1366 ± 94	142 ± 52	178 ± 59	206 ± 53	220 ± 61
Mangafodipir + diltiazem	1219 ± 69	96 ± 34	128 ± 42	158 ± 66	177 ± 24

Healthy rats (group sizes *n*=4 unless otherwise stated) administered MnCl_2_ (22 *μ*mol/kg), EVP1001-1 (22 *μ*mol/kg), or mangafodipir (44 *μ*mol/kg) over 3-4 min with simultaneous administration of 0.9% saline or diltiazem (100–200 *µ*mol/kg/min) infusion. Note the approximate 30% reduction in mean myocardial T_1_ values at each time point, but that there is greater discrimination between the diltiazem and saline infused rats due to the superior T_1_ shortening with EVP1001-1. Post hoc Bonferroni multiple comparisons (manganese agent + saline; manganese agent + diltiazem). Significance *P* < 0.05 at each time point as compared with saline control is indicated by asterisk.

**Table 2:** Mean LVEF and mean difference in LVEF versus baseline for the cohort of healthy rats administered MnCl_2_, EVP1001-1, and mangafodipir.

Time point (minutes)	Mean LVEF (%) and mean difference in LVEF (%)			
	Baseline	15	30	50
MnCl_2_ + saline	67.5 ± 5.6	73.4 ± 6.3	72.4 ± 5.6	71.1 ± 2.0
Mean difference (vs. baseline)	—	5.8 ± 1.3^*∗*^	4.9 ± 2.6	3.6 ± 5.1^*∗*^
MnCl_2_ + diltiazem	65.0 ± 8.0	58.2 ± 3.9	58.3 ± 6.8	60.1 ± 12.5
Mean difference (vs. baseline)	—	−3.2 ± 0.6^*∗*^	−3.1 ± 2.6^*∗*^	−4.8 ± 5.1^*∗*^
EVP1001-1 + saline	65.3 ± 0.7	71.0 ± 5.5	67.1 ± 2.5	67.1 ± 3.8
Mean difference (vs. baseline)	—	4.0 ± 6.8	−0.3 ± 2.6	0.7 ± 5.5
EVP1001-1 + diltiazem	69.6 ± 6.4	68.3 ± 8.4	63.4 ± 7.3	65.6 ± 7.8
Mean difference (vs. baseline)	—	−0.5 ± 3.7	−4.1 ± 4.0	−4.1 ± 3.7
Mangafodipir 22 *μ*mol/kg	63.2 ± 6.3	66.9 (*n*=2)	65.8 ± 8.2	67.1 ± 7.4
Mean difference (vs. baseline)	—	6.9 (*n*=2)	4.7 ± 7.1	2.5 ± 4.5
Mangafodipir 44 *μ*mol/kg + Saline	73.5 ± 3.1	76.8 (*n*=2)	76.1 ± 4.9	77.0 ± 4.1
Mean difference (vs. baseline)	—	3.9 (*n*=2)	4.5 ± 1.1	4.6 ± 3.6
Mangafodipir 44 *μ*mol/kg + diltiazem	64.1 ± 4.8	64.0 ± 8.1	62.5 ± 7.3	62.0 ± 9.5
Mean difference (vs. baseline)	—	0.81 ± 6.53	−0.2 ± 6.9	−1.0 ± 4.7

Group sizes *n*=3 for EVP1001-1 and mangafodipir 22 *μ*mol/kg and *n*=4 for MnCl_2_ and mangafodipir 44 *μ*mol/kg. Calculation of mean only from those time points with *n*=2 measurements available. Post hoc Bonferroni multiple comparisons significance *P* < 0.05 at each time point as compared to saline control indicated by asterisk.

## Data Availability

The data used to support the findings of this study may be made available from the corresponding author upon request.

## Abbreviations

DEMRI: Delayed enhancement magnetic resonance imaging
DPDP: Dipyridoxyl diphosphate
ETL: Echo train length
FOV: Field of view
MEMRI: Manganese-enhanced magnetic resonance imaging
MoLLI: Modified Look-Locker inversion recovery
MRI: Magnetic resonance imaging
MTC: Masson's trichrome
NMR: Nuclear magnetic resonance
ROI: Region of interest
TE: Echo time
TR: Repetition time.

## Supplementary Materials

Supplementary Materials S1: normalisation of T_1_ maps. S2: inter-/intraobserver variability studies for myocardial ROI contouring methodology.
