# Supplementary material for: Manganese-Enhanced T1 Mapping in the Myocardium of Normal and Infarcted Hearts
Source: Contrast Media Mol Imaging. 2018 Oct 25;2018:9641527. doi: 10.1155/2018/9641527 (PMC6222240; doi:10.1155/2018/9641527)
Supplement: Supplementary Materials — S1: normalisation of T1 maps. S2: inter-/intraobserver variability studies for myocardial ROI contouring methodology. [file 9641527.f1.zip › 9641527.f1/Preclinical MEMRI Supplemental Material v2.2.docx]

**Supplemental Material**

**S1. Normalisation of T1 Maps**

**Rationale**

During the lengthy *in vivo* experiments it was noted that there was an approximate ±10% variation in the measurement of T1 values in healthy myocardium and skeletal muscle before the administration of manganese contrast agents. It was found that this was due to beat-beat variation while under anaesthesia (and later chronotropic manganese contrast agents) during the 10-12 min required for to perform the T1 mapping using the pre-clinical MoLLI sequence. In an effort to compensate for this effect and to provide reliable data from which potentially small differences in regional T1 values could be studied (particularly in the infarct model), the T1 maps were normalised between baseline and at each subsequent time point post-administration of the manganese contrast agent using the T1 of the skeletal muscle. The experiments detailed below were designed to ensure that the skeletal muscle T1 did not vary over the time course of the experiments after manganese administration and therefore demonstrate that it was an acceptable tissue to use for normalisation of the myocardial T1 values. To this end two experiments were performed: 1) delivery of a bolus of higher molar dose of manganese to establish that the skeletal muscle T1 values did not change significantly and 2) imaging with an external phantom with a known T1 placed into the field of view to act as a reference T1 value to demonstrate that the skeletal muscle T1 values are representative as a reference tissue and are unchanged over the time-course of the experiments.

**Methods**

**Delivery of Large Manganese Bolus to a Healthy Rat**

The male Sprague Dawley rat (age 77 days, weight 325g) was anaesthetized with isofluorane and prepared as described in the main body of the paper with the tail vein cannulated and continuous monitoring of the heart rate, respiration rate and rectal temperature.

Imaging was performed using the experimental apparatus and methods detailed in the main body of the paper with scout images taken for localisation of the mid-ventricular short axis slice for interrogation with the MoLLI T1 mapping sequence.

Manganese chloride solution (MnCl2) was prepared as previously described but administered as 44 μmol/kg manganese in a volume of 2.2 mL/kg, diluted with 0.9% saline solution over 3-4 minutes with a further saline flush of 0.4 mL to ensure complete delivery to the circulation through the fine bore polyvinylchloride (PVC) long line.

**External Phantom Used During Manganese Enhanced MRI of Healthy Rat Myocardium Experiments**

A phantom was created using a loop of hollow PVC UN880 oxygen bubble tubing (Universal Hospital Services Inc., Minneapolis, USA) containing 529 mg/mL gadolinium complex (gadobendate dimeglumine, Bracco S.p.A, Milan, Italy) diluted to 0.2% with sterile water. The phantom T1 was measured using a fast spin echo IR-prepared T1 mapping sequence with the following parameters: TR=5s; TE=5.2ms; FOV=60x60mm2; matrix=128x128; ETL=16, slice thickness=5 mm, 8 equally spaced inversion times ranging from 0.01 to 4.00 s. The measured T1 of the phantom was 1320.0 ms.

The male Sprague Dawley rat (age 170 days and weight 460 g) was anaesthetized with isofluorane and prepared as described in the main body of the paper with the tail vein cannulated and continuous monitoring of the heart rate, respiration rate and rectal temperature. The external phantom was wrapped around the thorax of the rat before positioning in the cradle to provide an external reference.

Imaging was performed using the experimental apparatus and methods detailed in the main body of the paper with scout images taken for localisation of the mid-ventricular short axis slice for interrogation with the MoLLI T1 mapping sequence.

EVP1001-1 was administered as an intravenous bolus at a dosage of 22 μmol/kg manganese over 3-4 min as a volume of 2.2 mL/kg, diluted with 0.9% saline solution with a further saline flush of 0.4 mL to ensure complete delivery to the circulation from the long fine bore PVC line.

**Image Analysis and Normalisation Procedure**

As described in the main paper, the 14-20 images at unique inversion times were combined to generate T1 maps using CVI4.2® (Circle Cardiovascular Imaging, Calgary, Canada). For normalisation, the skeletal muscle T1 values at each time point () were calculated from ~10 mm2 regions of interest (ROI) drawn on the chest wall. A baseline skeletal muscle T1 value, , was similarly measured from identically situated ROIs. The ratio of skeletal muscle T1 values between each time point and baseline () were then used to normalise the T1 maps at each time point after the baseline images as follows:

[Eq. S1]

where is the absolute T1 of each voxel and is the normalised T1 of each voxel. Final normalised T1 maps were then generated in Matlab (MathWorks Inc., USA) with normalised T1 values obtained from ROIs drawn on the left ventricular myocardium at baseline before and after manganese contrast agent administration.

Absolute and normalised T1 maps at each time point were then analysed with regional T1 values of the phantom, skeletal muscle on the chest wall, blood pool in the left ventricle, and the left ventricular myocardium measured from ROIs. Values expressed represent mean ± standard deviation. Data are presented as mean ± standard deviation unless otherwise stated.

**Results and Discussion**

**Delivery of Large Manganese Bolus to a Healthy Rat**

Absolute T1 mapping data from the rat administered the 44 μmol/kg bolus of MnCl2 demonstrates that there was little change in the skeletal muscle T1 while there was a notable reduction in T1 in the myocardium and the blood pool (Figure S1-1A). The variation of T1 over time following administration of MnCl2, in comparison to skeletal muscle, is demonstrated (Figure S1-1B). There was noted to be little change in the skeletal muscle T1 during the experimental timeframe with a mean T1 of the skeletal muscle = 1236±39ms across all time points, while the blood pool T1 showed considerable variation (mean T1 of the blood pool = 1052±148ms). It should be noted that during the experiment, the heart rate of the rat was remarkably stable.

**Figure S1-1. Absolute T1 maps of healthy rat infused with 44 μmol/kg of manganese chloride solution.**

**A.** Absolute T1 maps of data acquired from a healthy rat administered with intravenous manganese chloride solution (MnCl2, 44 μmol/kg) over 3-4 minutes. Region of interests (ROIs) drawn around 1) skeletal muscle on anterior chest wall, 2) left ventricular blood pool and 3) left ventricular myocardium. Subsequent T1 map values were calculated from the same ROIs with all values expressed as mean ± standard deviation. **B.** Graph of the absolute mean T1 values for each of the ROIs indicated in S1-1A plotted against time, with two successive baseline values pre-EVP1001-1 administration.Note the large drop with recovery in T1 values of the blood pool over the timeframe with minimal change in the skeletal muscle values.

**External Phantom Used During Manganese Enhanced MRI of Healthy Rat Myocardium Experiments**

Absolute and normalised T1 mapping data from the rat administered 22 μmol/kg bolus EVP1001-1 with the external phantom in situ are shown in Figure S1-2. It can be seen that there was a ~100ms fluctuation in the measurement of the phantom and skeletal muscle T1 at baseline (Figure S1-2A Baseline 1 and Baseline 2) which would have made interpretation of the change in T1 values after EVP1001-1 administration difficult. It can be seen that the T1 values of the skeletal muscle follow those of the phantom in S1-2C and, unlike the blood pool, would not be susceptible to variation in the initial period after the manganese bolus as demonstrated in Figure S1-1.

As it would be expected that the ratio would be unity if there were no fluctuations in the measured skeletal muscle T1 values between experiments in the same animal at each time point, any deviation therefore provides a correction factor for normalisation. This simple normalisation is similar to that performed elsewhere [[[1]](#endnote-1),[[2]](#endnote-2)] and provides a pixel-wise map representing the T1 value at each spatial location. Using this normalisation procedure it was therefore possible to establish a relatively consistent skeletal muscle and phantom T1 during these experiments (Figure S1-2B), which allowed for closer inspection of the change in myocardial and blood T1 values.

**Figure S1-2. Absolute and normalised T1 maps of the healthy rat thorax with external phantom present.**

**A.** Raw T1 map data acquired from healthy rat with external phantom (described in text) wrapped around thorax and administered with intravenous EVP1001-1 (22 μmol/kg) over 3-4 minutes. Region of interests (ROIs) drawn around 1) external phantom, 2) skeletal muscle on anterior chest wall, 3) left ventricular blood pool and 4) left ventricular myocardium. Subsequent T1 map values were calculated from the same ROIs with all values are expressed as mean ± standard deviation. **B.** Baseline and normalised T1 maps produced from the T1 maps in A. with values calculated from the ROIs indicated in baseline image 1 of S1-2A. Note that the blood pool values. **C.** Graph of the absolute and normalized T1 values for different tissues over time, compared to phantom data, with two successive baseline values pre-EVP1001-1 administration.

**S2. Inter- /Intra-observer variability studies for myocardial ROI contouring methodology**

**Rationale**

As a result of the variation in T1 values described above, and in the context of no existing standardised methodology for quantifying infarct with T1 mapping in rodents, an investigative approach was adopted. Standardised methods have previously been explored to adjust the thresholding of automated late-enhancement detection software applying between 2 and 6 standard deviations of a mean of remote myocardial signal intensity to allow quantification of late-enhancement [[[3]](#endnote-3),[[4]](#endnote-4)]. To mitigate against the propensity to over-estimate infarct size using this technique, the same quantification approach was applied to the infarct regions, providing a quantitative methodology for compiling a T1 colour map, whereby visual discrimination was straight-forward based on pixel-wise colour differential. This same methodology applied to all T1 maps, for both agents, and both time-points.

Although not proposed for clinical translation and necessary only a direct result of the preclinical model used in this study, the exploratory nature of this methodology calls for inter- and intra-observer variability data for reinforcement.

**Methods**

Three T1 maps were acquired for each of thirteen infarcted animals for each time point. A varied sample of 6 was selected to represent approximately 15% of T1 maps from one time point, representative of native (n=2), DEMRI (n=2) and MEMRI (n=2) T1 maps, from animals with different left ventricular ejection fraction. T1 maps were reported by 2 independent operators, both experienced in cardiac MRI and image analysis software. Each T1 map was analysed on two separate occasions by each operator. Five key T1 mapping quantification parameters were reported for comparison; mean myocardial T1, mean remote T1, mean infarct T1, remote myocardium area and infarct area. Inter-operator agreement was assessed with Bland-Altman plots and Pearson correlation. Intra-operator variability assessed with paired t-test.

**Results and Discussion**

Bland-Altman plots demonstrate excellent agreement between operators with all five T1 mapping parameters showing only small degrees of bias and 95% confidence intervals of less than 10% (Table 1, Figure S2-1 A), showing strongly positive correlation between operators (Figure S2-1 B). Given the complexity of the experimental protocols in this study, scan-rescan repeatability studies were not feasible but the inter- and intra-operator variability studies suggest the methodology is robust and repeatable.

| **Parameter** | **Bias (% change)** | **95% CIs** |
| --- | --- | --- |
| Mean T1 whole myocardium | 0.07 | -1.6 to 1.7 |
| Mean T1 remote myocardium | -0.04 | -1.3 to 1.2 |
| Mean T1 infarct | -2.18 | -7.9 to 3.6 |
| Remote area | -2.05 | -4.7 to 0.6 |
| Infarct area | 0.02 | -9.9 to 9.9 |

Table S2-1. Inter-operator agreement between operators for 5 key parameters of myocardial contouring by Bland-Altman.

|  | **Parameter** | **Mean % change (SD)** | **P value** |
| --- | --- | --- | --- |
| Operator 1 | Mean T1 whole myocardium | 0.20 (0.02) | 0.6 |
|  | Mean T1 remote myocardium | 4.42 (0.07) | 0.3 |
|  | Mean T1 infarct | 0.18 (0.01) | 0.5 |
|  | Remote area | 2.98 (0.15) | 0.6 |
|  | Infarct area | -1.89 (0.04) | 0.7 |
| Operator 2 | Mean T1 whole myocardium | -0.26 (0.01) | 0.5 |
|  | Mean T1 remote myocardium | -1.62 (0.02) | 0.2 |
|  | Mean T1 infarct | -2.13 (0.04) | 0.4 |
|  | Remote area | 0.91 (0.10) | 0.7 |
|  | Infarct area | -6.97 (0.12) | 0.2 |

Table S2-2. Intra-operator agreement between operators for 5 key parameters of myocardial contouring.

**Figure S2-1. Inter-operator agreement across 5 T1 mapping parameters for 6 T1 maps.**

**A.** Bland-Altman plots demonstrating excellent agreement with narrow confidence intervals.

**B.** Pearson correlation showing strongly positive correlation between operators for all T1 mapping parameters.

**References**

1. . Hu TC, Pautler RG, MacGowan GA, & Koretsky AP. Manganese-enhanced MRI of mouse heart during changes in inotropy. Mag Reson Med. 2001; 46(5), 884-890. [↑](#endnote-ref-1)
2. . Dasenbrook EC, Lu L, Donnola S, Weaver DE, Gulani V, Jakob PM, et al. Normalized T1 Magnetic Resonance Imaging for Assessment of Regional Lung Function in Adult Cystic Fibrosis Patients - A Cross-Sectional Study. PLoS One. 2013;8(9), e732866. [↑](#endnote-ref-2)
3. Bondarenko O, Beek AM, Hofman MB, Kühl HP, Twisk JW, van Dockum WG, et al. J Cardiovasc Magn Reson. Standardizing the definition of hyperenhancement in the quantitative assessment of infarct size and myocardial viability using delayed contrast-enhanced CMR. 2005;7(2):481-5. [↑](#endnote-ref-3)
4. West AM, Kramer CM. Cardiovascular magnetic resonance imaging of myocardial infarction, viability, and cardiomyopathies. Curr Probl Cardiol. 2010;35(4):176-220. [↑](#endnote-ref-4)
